# Long-term persisting hybrid swarm and geographic difference in hybridization pattern: genetic consequences of secondary contact between two *Vincetoxicum* species (Apocynaceae–Asclepiadoideae)

**DOI:** 10.1186/s12862-016-0587-2

**Published:** 2016-01-22

**Authors:** Yue Li, Fumito Tada, Tadashi Yamashiro, Masayuki Maki

**Affiliations:** Botanical Gardens, Tohoku University, Sendai, Aoba 980-0862 Japan; Division of Ecology and Evolutionary Biology, Graduate School of Sciences, Tohoku University, Sendai, Aoba 980-8578 Japan; Department of Life Science, Faculty of Integrated Arts and Sciences, The University of Tokushima, Minami-jyosanjima, Tokushima, 770-8502 Japan

**Keywords:** Historical introgression, Hybrid swarm, Nuclear microsatellite, Past secondary contact, *Vincetoxicum*

## Abstract

**Background:**

During glacial periods, glacial advances caused temperate plant extirpation or retreat into localized warmer areas, and subsequent postglacial glacial retreats resulted in range expansions, which facilitated secondary contact of previously allopatric isolated lineages. The evolutionary outcomes of secondary contact, including hybrid zones, dynamic hybrid swarm, and resultant hybrid speciation, depends on the strengths of reproductive barriers that have arisen through epistatic and pleiotropic effects during allopatric isolation. The aim of this study was to demonstrate refugia isolation and subsequent secondary contact between two perennial Asclepioid species and to assess the genetic consequences of the secondary contact. We modeled the range shift of two ecologically distinct *Vincetoxicum* species using the species distribution model (SDM) and assessed the genetic consequences of secondary contact by combining morphological and genetic approaches. We performed morphometric analysis (592 individuals) and examined 10 nuclear microsatellites (671 individuals) in *V. atratum*, *V. japonicum*, and putative hybrid populations.

**Results:**

Multivariate analysis, model-based Bayesian analysis, and non-model-based discriminant analysis of principal components confirmed the hybridization between *V. atratum* and *V. japonicum*. High pollen fertility and a lack of linkage disequilibrium suggested that the hybrid populations may be self-sustaining and have persisted since *V. atratum* and *V. japonicum* came into contact during the post-glacial period. Moreover, our findings show that the pattern of hybridization between *V. atratum* and *V. japonicum* is unidirectional and differs among populations. Geographically-isolated hybrid populations exist as genetically distinct hybrid swarms that consist of *V. atratum*-like genotypes, *V. japonicum*-like genotypes, or admixed genotypes. In addition, Bayesian-based clustering analysis and coalescent-based estimates of long-term gene flow showed patterns of introgressive hybridization in three morphologically ‘pure’ *V. japonicum* populations.

**Conclusion:**

In this study, we demonstrated that climatic oscillations during the Quaternary period likely led to species range shift and subsequently secondary contact. Hybrid populations may be self-sustaining and have persisted since *V. atratum* and *V. japonicum* came into contact during the post-glacial period. Pattern of hybridization between *V. atratum* and *V. japonicum* is unidirectional and differs among populations. We concluded that these differences in the genetic consequences of secondary contact are caused by historical colonization processes and/or natural selection.

**Electronic supplementary material:**

The online version of this article (doi:10.1186/s12862-016-0587-2) contains supplementary material, which is available to authorized users.

## Background

In response to climatic oscillations over the last 2 million years (Ma), the geographical distributions of temperate plants have been subject to multiple contractions and expansions [1]. During glacial periods, glacial advances caused temperate plant extirpation or retreat into localized warmer areas, and subsequent postglacial glacial retreats resulted in range expansions, which facilitated secondary contact of previously allopatric isolated lineages [2–4]. The evolutionary outcomes of secondary contact, including hybrid zones, dynamic hybrid swarm, and resultant hybrid speciation [5], depend on the strengths of reproductive barriers [6].

Assuming that the reproductive isolation between two species coming into contact was incomplete, first generation hybrids may mate with parental species. Frequently backcrossing between hybrids and their parental species will lead introgression of alleles across species boundaries, thereby affecting species history and delimitation [7]. The dynamics of introgression for neutral alleles are thought to be depends on the reproductive system of the interacting taxa [8–11], but also on relative population abundance [12]. Genes are assumed to flow from large populations of one species into much smaller populations. Recent theoretical models predicted that the direction of introgression of neutral alleles between lineages should proceed from the locally established species towards the expanding congener [13, 14]. In addition, genotype combinations might be filtered by selection factors that vary with the surroundings (genotype-by-environment interactions) [15–22].

In recent years, an increasing number of studies have employed species distribution modeling (SDM) or ecological niche modeling (ENM) to support refugia isolation and subsequent range expansion and corroborate phylogenetic approaches. The aim of this study was to demonstrate refugia isolation and subsequent secondary contact by modeling range shifts. This study presents one of the first applications of the species distribution model (SDM) to explain the direction of historical gene flow and its effects on the genetic consequences of secondary contact by predicting potential glacial distribution and postglacial colonization processes.

The Japanese Archipelago is thought to have been free of massive ice sheets during the last glacial maximum (LGM) with the exception of some high altitude areas [23]. Previous phylogenetic studies showed that the postglacial range expansion process may have caused secondary contact between formerly isolated lineages of *Fagus crenata* [24], *Aesculus turbinata* [25], *Padus grayana*, *Euonymus oxyphyllus*, *Magnolia obovata*, *Carpinus tschonoskii*, *C. japonica*, *C. laxiflora* [26], *Platycrater arguta* [27], *Rubus palmatus,* and *R. grayanus* [28]. Two ecologically distinct species of *Vincetoxicum* (Apocynaceae-Asclepiadoideae), *V. atratum* (Bunge) Morren et Decaisne and *V. japonicum* Morren et Decne., provide a suitable study system. Because the different habitat preferences of the species may result in different responses to climate change [3, 29–31], *V. atratum* and *V. japonicum* may have retreated into separate refugia during the glacial period and subsequent postglacial expansion may have facilitated secondary contact, resulting in hybridization.

*Vincetoxicum atratum* and *V. japonicum* are perennial herbs. *Vincetoxicum atratum* is distributed across the Sino-Japanese region including the Japanese Archipelago, Korean Peninsula, and China, whereas *V. japonicum* is distributed in the Japanese Archipelago and the Korean Peninsula [32]. In Japan, *V. atratum* is found in grasslands or meadows across northern to southern regions of the archipelago, while *V. japonicum* is found in sunny meadows or on rocky beaches near seashores of central and southern regions of the archipelago [32]. Because the habitat area has diminished due to habitat destruction and degradation, the number and sizes of *V. atratum* populations have decreased rapidly. Morphologically, the two species can be distinguished based on specific characteristics. *Vincetoxicum atratum* has a purple corolla, which is pubescent outside and glabrous inside, while *V. japonicum* has a yellowish white corolla, which is glabrous outside and pubescent inside. Leaves of *V. atratum* are villous on both sides while those of *V. japonicum* are densely pubescent [32]. The flowering periods of these species partially overlap in early summer. Some dipteran species are pollinators of both *V. atratum* and *V. japonicum* [33]. The low level of genetic diversification in the chloroplast DNA (cpDNA) of the two species in contrast with the considerable morphological divergence suggests that the species have undergone rapid morphological divergence [34].

In this study, we examined past hybridization and introgression between *V. atratum* and *V. japonicum* by combining morphological and genetic approaches with paleoclimatic analysis. First, we examined the hybrid status of individuals from seven morphologically putative hybrid populations using a morphometric approach. Second, we examined the genetic structure of morphologically ‘pure’ *V. atratum*, *V. japonicum*, and putative hybrid populations. In particular, we examined deviation from linkage equilibrium for nuclear microsatellite markers because linkage disequilibrium would be expected in a hybrid zone as a result of ongoing gene flow between the parent species [35–37]. Third, we predicted species paleo-distributions in response to the Quaternary climate change to model range shifts of *V. atratum* and *V. japonicum* and to infer past opportunities for secondary contact between the species. In this report, we address the following three questions. (1) What are the genetic and phenotypic patterns of morphologically ‘pure’ *V. atratum*, *V. japonicum*, and putative hybrid populations? (2) What is the outcome of secondary contact between *V. atratum* and *V. japonicum*? (3) What is the genetic consequence of hybridization between the two species?

## Methods

### Study site and sampling

**Fig. 1:**
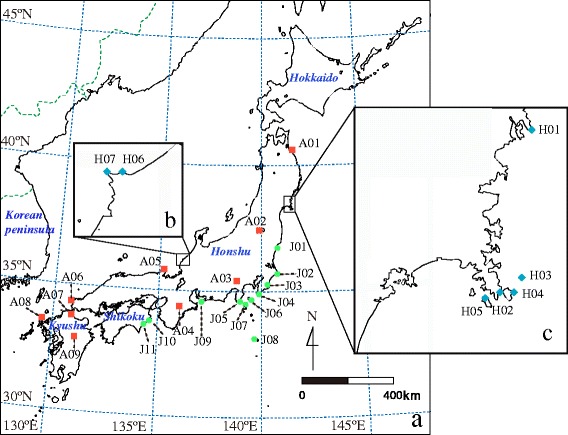
Maps of the hybrid zone between *V. atratum* and *V. japonicum* and of morphologically ‘pure’ *V. atratum* populations. **a** Map of Japan showing locations of the hybrid zone and morphologically ‘pure’ *V. atratum* populations (filled red cube) and *V. japonicum* populations (filled green circles). **b**, **c** Detailed map of the hybrid zones showing the putative hybrid sampling sites (blue diamond). Additional details for each population are shown in Additional file 1: Table S1

We sampled morphologically ‘pure’ populations of *V. atratum* and *V. japonicum* from almost the entire ranges of the two species in Japan (Fig. 1; Additional file 1: Table S1). Seven morphologically hybrid populations between *V. atratum* and *V. japonicum* were sampled (Fig. 1; Additional file 1: Table S1). Morphologically hybrid populations were tentatively identified based on the corolla color and vestiture on leaves. We did not detect morphologically ‘pure’ populations of *V. atratum* and *V. japonicum* near any of the tentative hybrid populations. Leaf samples of individuals, separated by at least 2 m, were collected to avoid resampling the same genotypes. The leaf samples were stored at −70 °C until DNA extraction.

### DNA extraction and nuclear microsatellite scoring

Total DNA was extracted from approximately 100 mg of leaf tissue based on the method of Maki, Horie and Yokoyama [38]. Ten nuclear simple sequence repeat (nrSSR) loci including vinc5, vinc107, vinc118 [39], Vpy 012, Vpy 013, Vpy 016, Vpy 018, Vpy 022 [40], Vkat3, and Vkat2 (Yamashiro et al., unpublished,) were scored. These 10 loci were amplified in three multiplex reactions (Additional file 1: Table S2)*.* Multiplex PCRs were performed in 3-μL volumes containing approximately 50 ng genomic DNA, 1 × Type-it Multiplex PCR Master Mix (QIAGEN), and 0.2 μM each of forward and reverse primers. The reaction parameters were an initial denaturation step at 95 °C for 5 min followed by 26 cycles at 95 °C for 30 s, 60 °C for 90 s, and 72 °C for 60 s, and a final step at 60 °C for 30 min. PCR products were run with an internal size standard GeneScan LIZ-600 (Applied Biosystems) on an ABI 3100 Genetic Analyzer (Applied Biosystems). Allele sizes were scored using GENEMAPPER v3.7 software (Applied Biosystems).

### Morphological measurements

**Fig. 2:**
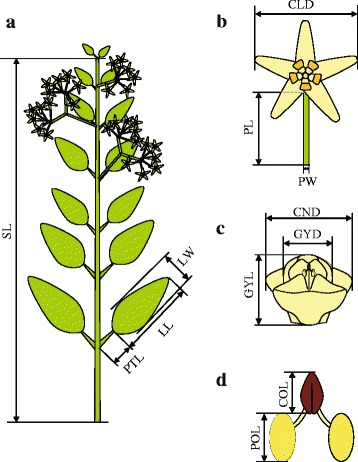
Schematic diagrams showing the morphological characteristics measured and used in the morphometric analyses

Morphological measurements were performed on the same individuals that were genotyped. The four largest mature leaves and four largest mature flowers of each individual were subjected to measurement of morphological characteristics and the averages of the measurements per individual were used for morphometric analyses. A total of 13 quantitative and four semi-quantitative characteristics were measured (Fig. 2; Table 1).

**Table 1:** Description of the morphological characteristics scored for morphologically ‘pure’ *V. atratum* and *V. japonicum* plants and morphologically intermediate plants, and the results of one way ANOVA or Kruskal-Wallis ANOVA of morphologically ‘pure’ *V. atratum* and *V. japonicum* plants

Morphological characteristic	Label	Trans.	Species F (dF = 1)
Maximum leaf blade length (cm)	LL	None	127.70***
Maximum leaf blade width (cm)	LW	None	34.89***
Petiole length (cm)	PL1	None	0.03
Stem length (cm)^a^	SL	Log	2.12
Hair density on adaxial leaf surface (cm)^b^	HDL	None	***
Hair density on abaxial leaf surface (cm)^c^	HBL	None	***
Number of flowers	NF	None	5.41*
Pedicel length (cm)	PL	None	5.08*
Pedicel width (cm)	PW	None	1371.00***
Corolla diameter (cm)	CLD	None	649.10***
Hair density on corolla outside (cm)^d^	HCO	None	***
Hair density on corolla inside (cm)^e^	HCI	None	***
Corona diameter (cm)	CND	None	1882.00***
Gynostegium length (cm)	GYL	None	2027.00***
Gynostegium diameter (cm)	GYD	None	2913.00***
Corpusculum length (cm)	COL	None	1154.00***
Pollinia length (cm)	POL	None	378.30***

^a^Trans. = transformations applied to achieve normality and homogeneity of variances (Log = Natural Logarithm)

^b^Ordinal grades 1–10. 1 = less than 20, 2 = 21–40, 3 = 41–60, 4 = 61–80, 5 = 81–100, 6 = 101–120, 7 = 121–140, 8 = 141–160, 9 = 161–180, and 10 = 181–200 (hairs/cm^2^)

^c^Ordinal grades 1–10. 1 = less than 40, 2 = 41–80, 3 = 81–120, 4 = 121–160, 5 = 161–200, 6 = 201–240, 7 = 241–280, 8 = 281–320, 9 = 321–360, and 10 = 361–400 (hairs/cm^2^)

^d^Ordinal grades 1–10. 1 = less than 10, 2 = 11–20, 3 = 21–30, 4 = 31–40, 5 = 41–50, 6 = 51–60, 7 = 61–70, 8 = 71–80, 9 = 81–90, and 10 = 91–100 (hairs/cm^2^)

^e^Ordinal grades 1–10. 1 = less than 10, 2 = 11–20, 3 = 21–30, 4 = 31–40, 5 = 41–50, 6 = 51–60, 7 = 61–70, 8 = 71–80, 9 = 81–90, and 10 = 91–100 (hairs/cm^2^)

* *P* < 0.05, ** *P* < 0.01, *** *P* < 0.001

### Morphometric analysis

For 13 continuous variables, significant differences between allopatric *V. atratum* and *V. japonicum* were tested using one-way analysis of variance (ANOVA). Pearson correlation coefficients were calculated to eliminate highly correlated (Pearson’s correlation > 0.9) characteristics from further analyses. A Kolmogorov-Smirnov test was used to determine whether the data met the assumption of normal distribution, while a Levene’s test was used to test for homoscedasticity. For four ordinal variables, Kruskal-Wallis one-way ANOVA was performed to estimate differences between *V. atratum* and *V. japonicum*. Principal coordinate analysis (PCO) was used to visualize morphological variation among morphologically ‘pure’ *V. atratum*, *V. japonicum* populations and morphologically hybrid populations. All statistical analyses were performed using R programming language [41].

### Pollen viability estimation

Pollen fertility was examined in three populations of *V. atratum* (A01–A03) and *V. japonicum* (J01–J03) and in all putative hybrid populations following the procedure of [42].

### Population genetic analysis

For each population, the mean number of alleles per locus (*N*_a_), observed heterozygosity (*H*_O_), and expected heterozygosity (*H*_E_) were calculated using the program GenAlEx version 6 [43]. Allelic richness (AR) and private allele richness (PAR) were estimated using HP-RARE 1.1 software [44]. Genetic differentiation in all pair wise combinations of the populations was estimated from the fixation index (*F*_ST_, [45] for nuclear simple sequence repeats (nrSSRs) with 1000 permutations using Arlequin version 3.5 software [46]. Tests for deviation from the Hardy-Weinberg equilibrium (HWE) at each locus were conducted with Arlequin version 3.5 software using 10,000 permutations [46]. Tests for the linkage disequilibrium (LD) of all combinations of the loci for each population were conducted using GENEPOP 4.2 software [47]. LD can be induced by genetic admixture between divergent gene pools [35, 36] and will decay if gene flow occurs between the divergent gene pools [37], thus, if no pair wise LD was detected among loci, we would expect that the hybrid population would not be maintained by ongoing gene flow.

### Bayesian admixture analysis

A Bayesian model-based approach implemented in the program STRUCTURE version 2.2.3 [48] and TESS version 2.3 software [49] was used to assess the genetic delimitation between *V. atratum* and *V. japonicum* and to estimate the proportion of each individual genome originating from each of the parental populations. We assigned individuals having an assignment value (*q*) greater than 0.9 into one of the parental lineages and classified those with values less than 0.9 as hybrids. For analysis using STRUCTURE, different probable numbers of genetic clusters (*K*) were estimated for all samples under the admixture model with correlated allele frequencies. Ten replicate runs were performed for each value of *K* ranging from 1 to 10. Each run included a burn-in of 10,000 steps followed by 100,000 Markov chain Monte Carlo (MCMC) simulations. The optimal value of *K* was calculated using the STRUCTURE HARVESTER program [50]. The results from the 10 replicates of the optimal value of *K* were averaged using the CLUMPP program [51] and were visualized using the DISTRUCT 1.1 program [52].

TESS version 2.3 was used to estimate population structure by incorporating geographical coordinates of individuals as prior information to detect discontinuities in allele frequencies [49]. The analyses were performed using a conditional autoregressive (CAR) model with a burn-in of 10,000 iterations followed by 60,000 iterations for each *K* value (the number of clusters) between 2 and 10. The optimal value of *K* was determined based on the lowest value of the deviance information criterion (DIC). The results from the 10 replicates with the lowest DIC values were averaged using CLUMPP [51] and were visualized using DISTRUCT 1.1 as in the STRUCTURE analyses [52].

### Discriminant analysis of principal components

To detect complex genetic structure, a non-model-based discriminant analysis of principal components (DAPC) using the adegenet version 1.2.8 package [53] in R [41] was conducted based on the SSR data set. The prior clusters were defined by the groups obtained from each population. DAPC first transforms genotype into uncorrelated components using principle component analysis (PCA) and then performs a discriminant analysis on the retained principle components (PCs).

### Estimation of long-term gene flow

We divided studied populations based on the genetic constitution of each population defined by STRUCTURE, namely, *‘*pure’ *V. atratum* (A01–A07 and A09), *‘*pure’ *V. japonicum* (J01–J08), south group of putative hybrid population (H01–H05), north group of putative hybrid population (H06–H07) and introgressed *V. japonicum* (J09–J11). We estimated bidirectional long-term migration rates among the five categories based on 10 nrSSR loci with a coalescent-based model implemented in the LAMARC 2.1 program [54]. We randomly picked genotype data for 20 individuals in each categories, because migrate history in additional individuals is probably similar to that of its cohorts. We estimated impacts of migration relative to mutation rate (*M* = *m*/μ) and the mutation-scaled effective population size (Θ = 4*N*_*e*_μ). *N*_*e*_ is the effective population size, *m* is the migration rate between two populations, and μ is the mutation rate per generation at the locus considered. We used logarithmic prior distributions for migration rate (0.01, 3000), and effective population size (0.00001, 10). We conducted Bayesian analyses under the Brownian motion model [55]. Search parameters consisted of 10 initial chains with 5,000 samples, a sampling interval of 30 (150,000 steps), and a burn-in of 10,000 samples; two final chains with 50,000 samples and a sampling interval of 30 (1,500,000 steps); and a burn-in of 200,000 samples. For each pair wise comparison (*i* → *j*), the number of immigrants from population *i* into population j (*N*_*e*_*m*_(*i* → *j*)_) was estimated from *N*_e_*m* = Θ_i_*M_(*i* → *j*)_ ⁄ 4, where Θ represents the mutation scaled effective population size (Θ = 4*N*_*e*_μ). Results are reported as most probable estimates (MPE) with 95 % confidence intervals (CIs). We used Tracer v1.6 [56] to confirm sampling adequacy and convergence of parameters. All parameter estimates were well supported, with ESS (effective sample size) values exceeding 200.

### Species distribution modeling

We used species distribution modeling (SDM) to identify the range of suitable habitats of *V. atratum* and *V. japonicum* during three periods, namely, the present, the mid-Holocene (6,000 ybp), and the last glacial maximum (LGM; 21,000 ybp). The maximum entropy algorithm (MAXENT, Phillips and Dudík; 2008), based on presence-only data, and the associated environmental covariates were used to model species distribution. Species occurrence data were collected from the S-Net data portal (http://science-net.kahaku.go.jp/) and our sampling locations (Additional file 1: Table S3). We restricted the spatial extent of the modeled area to only Japanese Archipelago for both species. SDM was constructed using information for 125 *V. atratum* and 128 *V. japonicum* sites to predict the geographic distributions of each species (Additional file 1: Table S3; Additional file 1: Figure S1). Collection coordinates were plotted in Google Earth (http://earth.google.com) to confirm that each set represented a reasonable location. A total of 19 environmental variables (Additional file 1: Table S4) were downloaded from the WorldClim website (www.worldclim.org; [57]. We extracted the values of each of the 19 environmental variables for all occurrence recorder points using the DIVA-GIS program [58] and examined pair wise correlations using R [41]. After evaluation, variables with low pair wise Pearson correlation coefficients (*r* < 0.70) were used for subsequent analyses (Additional file 1: Table S3). We used the simulation summaries of the Community Climate System Model version 4 (CCSM4) to create mid-Holecene and LGM climate data projections. The resolutions of current, mid-Holecene, and LGM climate layers were 30 arcsec, 30 arcsec, and 2.5 arcmin, respectively. The distributional model for each species was generated using MAXENT 3.3.3 k [59] and included 75 % of species records for training and 25 % for testing the model. We employed receiver operating characteristic (ROC) analysis to measure model performance [60]. The accuracy of each model prediction was tested by the area under the ROC curve (AUC) [61]. AUC scores range from 0 to 1, with a score greater than 0.7 considered to be a good model performance [62].

## Results

### Morphological differentiation

Hair density on the abaxial leaf surface (HBL), hair density on the adaxial leaf surface (HDL), and hair density on the corolla outside (HCO) were highly correlated with each other (*r* > 0.9), and thus HBL and HCO were eliminated from subsequent multivariate analyses. Three vegetative characteristics, maximum leaf blade length (LL), maximum leaf blade width (LW), and hair density on the adaxial leaf surface (HDL), and eight floral characteristics, pedicel width (PW), corolla diameter (CLD), hair density on the corolla inside (HCI), corona diameter (CND), gynostegium length (GYL), gynostegium diameter (GYD), corpusculum length (COL), and pollinia length (POL) that exhibited significant differences (*P* < 0.05) among morphologically ‘pure’ *V. atratum* and *V. japonicum* populations (Table 1) were used for multivariate analyses. Inter- and intra-population variations in these morphological characteristics are shown in box plots (Additional file 1: Figure S2) and bar plots (Additional file 1: Figure S3). The intra-population variation range for these characteristics varied among putative hybrid populations. For example, PW values of the H01 population tended to be similar to those of *V. atratum*, whereas the PW values of the H02–H05 populations tended to be similar to those of *V. japonicum*. The ranges of variation in PW in the H06 and H07 populations were intermediate between those of *V. atratum* and *V. japonicum*.

### Multivariate analysis

**Fig. 3:**
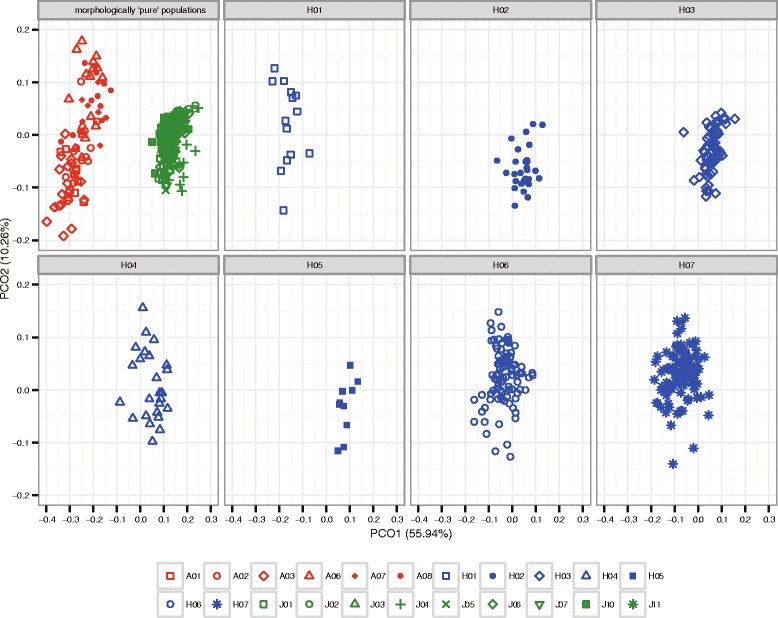
Principle coordinate analysis of 11 morphological characteristics of individuals from morphologically ‘pure’ *V. atratum* and *V. japonicum* plants and morphologically intermediate plants

The first and second axes in principal coordinate (PCO) analysis accounted for 55.94 and 10.26 % of the total variance, respectively (Fig. 3). The PCO analysis showed two distinct groups of clearly separating, morphologically ‘pure’ *V. atratum* and *V. japonicum* individuals along the first axis. In the putative hybrid populations, individuals of the H01 population were largely distributed over the range of variation of phenotypes of ‘pure’ *V. atratum*, as well as intermediate positions between those of the two species. Individuals of the H02 and H03 populations were largely distributed over the range of variation of phenotypes of ‘pure’ *V. japonicum*, as well as intermediate positions. Individuals of the H05 population overlapped with the range of variation of phenotypes of ‘pure’ *V. japonicum*. Individuals of the H04, H06, and H07 populations were mostly distributed over intermediate positions between the two species.

### Pollen fertility

The minimum, maximum, and median values of pollen fertility are summarized in Additional file 1: Table S5. All the populations examined exhibited high pollen fertility. Pollen fertility ranged from 97–100 % in *V. atratum* populations (A01–A03), 96–100 % in hybrid populations (H01–H07), and 100 % in *V. japonicum* populations (J01–J03).

### Microsatellite diversity

A total of 144 alleles were found for 10 nrSSR loci in *V. atratum* populations compared with 165 alleles for *V. japonicum*. The mean number of alleles per locus (*N*_a_) within a population was 5.30–8.10 in *V. atratum* and 2.60–8.90 in *V. japonicum* (Additional file 1: Table S1). The allele richness and the expected heterozygosity were 3.89–4.82 and 0.597–0.697, respectively, in *V. atratum*, and 2.06–4.34 and 0.289–0.667, respectively, in *V. japonicum* (Additional file 1: Table S1). While most of the loci were in Hardy-Weinberg equilibrium (HWE), 31 of 260 cases showed significant deviations from HWE expectation (Additional file 1: Table S6). While most of the locus pairs did not show significant deviation from linkage equilibrium, one pair of loci in the H06 and J02 populations was in LD after applying the Bonferroni correction (*P* > 0.01) (Additional file 1: Table S7). All pair wise *F*_ST_ estimates were significantly different from zero (*P*  <  0.05). Genetic differentiation between all pairs of *V. atratum* and *V. japonicum* populations were moderate or large with *F*_ST_ values ranging from 0.18 to 0.52 (Additional file 1: Table S8).

### Population genetic structure

**Fig. 4:**
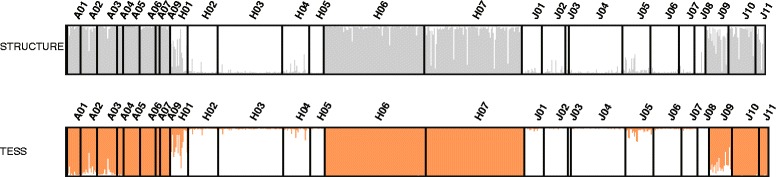
Admixture analysis showing the proportion of the genome of each individual originating from *V. atratum* or *V. japonicum* using the programs STRUCTURE and TESS. Each individual is represented as a vertical bar indicating the assignment score of each genetic group

**Fig. 5:**
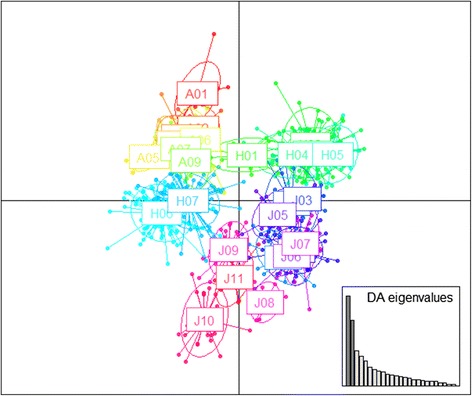
Discriminant analysis of principal components (DAPC) based on microsatellite data sets

The best value for the number of genetic clusters (*K)* determined from STRUCTURE analysis was 2 based on ∆*K* values (Additional file 1: Figure S4 and Fig. 4). The optimal *K* value determined using the TESS program was 5 based on the deviance information criterion (DIC). Because we focused on hybridization between the two species in this study, we adopted *K* = 2 *a priori*, even when using TESS. With *K* = 2, all morphologically ‘pure’ *V. atratum* populations and the J01–J08 *V. japonicum* populations clustered into two separate groups (Fig. 4). In contrast, most individuals of the morphologically ‘pure’ *V. japonicum* J09–J11 populations were assigned to *V. atratum* or considered admixed individuals. A large number of the individuals in the morphologically putative hybrid H02, H03, H04, and H05 populations were assigned to *V. japonicum*, whereas most of the individuals in the H06 and H07 populations were assigned to *V. atratum*. Many of the H01 individuals were considered to be admixed individuals (Fig. 4, Additional file 1: Table S9). Because of the relatively small number of microsatellite loci used, the Bayesian-based assignment approach may have assigned highly backcrossed individuals (which genetically resemble the parental form) to either of the parental categories. Discriminant analysis of principal components (DAPC) also identified all morphologically ‘pure’ *V. atratum* individuals and the *V. japonicum* J01–J08 populations as well-delimited genotype clusters (Fig. 5). Individuals of the introgressed *V. japonicum* populations (J09–J11) were distributed over intermediate positions near *V. japonicum*. In the H01, H06, and H07 putative hybrid populations, individuals were distributed over intermediate positions near *V. atratum*, whereas individuals of the H02–H05 populations were distributed over intermediate positions near *V. japonicum*.

### Long-term estimates of gene flow

Coalescent-based estimates obtained from the LAMARC program indicated that 4 *Nm*_‘pure’*V. atratum* →‘pure’ *V. japonicum*_ (19.27) was much higher than 4 *Nm*_‘pure’ *V. japonicum*→‘pure’*V. atratum*_(1.18) (Additional file 1: Table S10). In addition, there was no evidence of unequal amount genetic input into the putative hybrid population, because of overlapping CIs for estimates of 4 *Nm* in both directions.

### Species distribution modeling

**Fig. 6:**
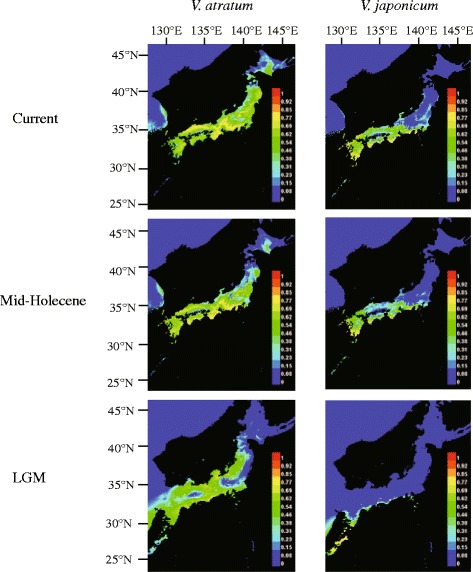
Predicted distributions of *V. atratum* and *V. japonicum* based on species distribution modeling using the MAXENT program. Predicted distributions are shown for the present time, the mid-Holocene, and the last glacial maximum (LGM)

SDM based on Community Climate System Model version 4 (CCSM4) data at the time of the LGM is shown in Fig. 6. SDM for both *V. atratum* and *V. japonicum* had high predictive performance with AUC values of 0.995 and 0.997, respectively. The predicted current potential distribution range was mostly consistent with known field observations and herbarium records in Japan. The potential ranges of both species in Japan during the LGM were apparently contracted compared to the present ranges. During the LGM, *V. atratum* was distributed mainly in the entire western part of Japan and along the Japan Sea side of Honshu. After the LGM, suitable habitats for *V. atratum* expanded gradually across most of Honshu. In addition, during the LGM, *V. japonicum* was restricted to possible southern refugia, mainly at the southern tip of Kyushu and in a narrow coastal belt of the Pacific side of Honshu. After the LGM, *V. japonicum* subsequently expanded its range across western Japan and some parts of eastern Japan. In addition, the predicted distribution ranges of *V. atratum* and *V. japonicum* in the Japanese Archipelago for the mid-Holocene did not differ substantially from the present distribution ranges.

## Discussion

### Shifts in the ranges of V. atratum and V. japonicum with climate change

The potential ranges of *V. atratum* and *V. japonicum* predicted by SDM suggested that these two ecologically distinct species responded to climate changes in different ways across the Japanese Archipelago (Fig. 6). During the LGM, *V. atratum* was distributed mainly in the entire western part of Japan and along the Japan Sea side of Honshu, whereas *V. japonicum* was restricted to possible southern refugia, mainly at the southern tip of Kyushu and in a narrow coastal belt of the Pacific side of Honshu. During the warmer post-glacial period, *V. atratum* expanded its range across most of the territory of Honshu, whereas *V. japonicum* expanded its range across western Japan and some parts of eastern Japan.

### Consequences of secondary contact and patterns of hybridization between V. atratum and V. japonicum

Morphological data confirmed the putative hybridization between *V. atratum* and *V. japonicum*. Morphologically ‘pure’ *V. atratum* and *V. japonicum* were readily discriminated based on the PCO analysis (Fig. 3) and variations in composite traits (Additional file 1: Figure S2–S3). All putative hybrid populations exhibited intermediate and/or parental-like phenotypes; however, morphological investigation alone is insufficient to explain gene flow-mediated hybridization patterns [63, 64]. We also used microsatellite markers to examine the genetic structure of hybrid populations. The Bayesian-based clustering analysis of nuclear microsatellite markers using STRUCTURE showed that the optimal value of *K* was 2 corresponding to the two morphologically ‘pure’ *V. atratum* populations and part of the morphologically ‘pure’ populations of *V. japonicum* (J01–J08) (Fig. 4). In the putative hybrid populations, most individuals exhibited *V. atratum*-like genotypes (H06 and H07), *V. japonicum*-like genotypes H02–H05 and admixed genotypes (H01). The DAPC of all individuals again showed two genetically distinct groups as did analysis based on STRUCTURE (Fig. 5). The H01, H06, and H07 populations were genetically closer to *V. atratrum*, whereas the H02–H05 populations were genetically closer to *V. japonicum*.

Most hybrid populations (H02–H05 and H06–H07) consist with parental-like genotypes. These genotypes may be generated by frequent backcrossing to one of parental species, because highly backcrossed individuals are difficult to distinguish from parental individuals due to the relatively small number of loci [65]. Unidirectional backcrossing might be explained by reproductive system [8–11] and/or relative species abundance [12]. Following reasons are possible to explain the fact. First, factors driving unidirectional backcrossing in plants usually include differences in flower structure [8], flowering time [9] and mating system [10] among interfertile species, differences in the offspring fitness of reciprocal crosses [12]. In the present study, there is no difference in flower structure, flowering time and mating system between *V. atratum* and *V. japonicum*. In addition, biases in offspring fitness unlikely exist, because both *V. atratum*-like genotypes (H06 and H07), *V. japonicum*-like genotypes H02–H05 and admixed genotypes (H01) were detected. Alternatively, the unidirectional backcrossing might result from ‘pollen swamping’ by more abundant local species over later-colonizing species [66–68]. The colonizing species may have been subject to substantial pollen flow from the local species and repeated backcrosses with incoming pollen would generate abundant backcrossing to the local aboriginal species. Genetic constitutions of hybrid populations H06 and H07 can be explained by the prediction of colonization process (Fig. 6); during the LGM, *V. atratum* were already distributed in the area where the current H06 and H07 populations are located. After the glacial period, *V. japonicum* may have colonized this area from its southern refugia and been subject to substantial pollen flow from *V. atratum*. If backcrosses to *V. atratum* are selectively favored and do not exhibit reduced fitness relative to *V. japonicum* and *V. atratum*, they may ultimately displace ‘pure’ populations of *V. japonicum* and *V. atratum*.

Although the SDM results indicated that *V. atratum* colonized the area where the current H01–H05 populations were located earlier than *V. japonicum* (Fig. 6), the genetic constitutions of the H01–H05 populations differed from those of the H06 and H07 populations (Fig. 5). We consider that the genetic constitutions of these hybrid populations between two *Vincetoxicum* were influenced not only by the historical colonization process, but were also regulated by other factor, such as, abiotic environmental regimes. Variation in fine-scale habitats might promote differences in the strengths of selection on recombination genotypes and lead to variable spatial structure in a narrow region [15–22]. In the present study, population H01 show different genetic constitution with population H02-H05. During field investigation, we found hybrid populations H01 located in high cliff, whereas population H02-H05 located in rocky beaches. Thus, population H01 and H02–H05 may have suffered different ecological selection and show different genetic constitution.

### Long-term hybrid swarm persistence

Hybrid swarms are believed to persist through two mechanisms [5, 69]. They may be maintained by frequent and strong gene flow that counterbalances selection against the hybrids. Alternatively, they may be self-sustaining and maintained largely by selective advantage. The hybridization between the two *Vincetoxicum* species undoubtedly falls into the latter circumstance. First, LD was observed in almost no combinations of loci in the hybrid populations (Additional file 1: Table S7) suggesting that the hybrid populations are not maintained by ongoing genetic input from parental populations and may have persisted for several generations. These attributes are characteristic of a hybrid swarm where the hybrids are self-sustaining [70]. Second, the pollen fertility of all individuals in the hybrid populations was very high (Additional file 1: Table S5) providing high viability to facilitate mating between the individuals. Combined with the SDM results (Fig. 6), which predicted probable secondary contact during the post-glacial period, one possible scenario to account for the existence of the hybrid swarm is that the hybrid populations probably formed when *V. atratum* and *V. japonicum* came into contact during the post-glacial period and may be self-sustaining and have persisted for a long term **–** since *V. atratum* and *V. japonicum* came into contact during the post-glacial period.

### Historic introgression

The Bayesian-based clustering analyses showed signs of introgressive hybridization (Fig. 5). In the J09–J11populations, the morphologically ‘pure’ *V. japonicum* population was composed of genetic clusters of both *V. atratum* and *V. japonicum*. Coalescent-based estimates from LAMARC also indicated high historic gene flow among ‘pure’ *V. atratum* and ‘pure’ *V. japonicum* populations (Additional file 1: Table S10). The modeled distribution shows that during LGM, suitable habitat for *V. japonicum* J09–J11 populations still persist in where they exist currently. There would have been a situation that small *V. japonicum* populations continued to persist in the area (where J09-J11 are located currently) surrounded by much larger populations of *V. atratum*, resulting in directional introgression from the latter species.

### Discrepancy in morphological and genetic identification of hybridity

The morphologies of individuals in the H06 and H07 hybrid populations were intermediate between those of *V. atratum* and *V. japonicum* (Fig. 3 and Additional file 1: Figure S2). Surprisingly, almost no individuals exhibited intermediate genotypes based on the results of microsatellite analyses (Fig. 4). A probable cause of this discrepancy is that morphology may be controlled by numerous genes, whereas a relatively small number of microsatellite loci were employed in the Bayesian-based assignment approach. Thus, microsatellite genotypes may be rapidly fixed to one of the parental species genotypes by backcrossing. However, the genes affecting morphology may have persisted over longer periods of time.

## Conclusions

In this study, we demonstrated that climatic oscillations during the Quaternary period likely led to species range shift and subsequently secondary contact. We confirmed the hybridization between *V. atratum* and *V. japonicum*. Hybrid populations may be self-sustaining and have persisted since *V. atratum* and *V. japonicum* came into contact during the post-glacial period. Moreover, our findings show that the pattern of hybridization between *V. atratum* and *V. japonicum* is unidirectional and differs among populations. In addition, Bayesian-based clustering analysis and coalescent-based estimates of long-term gene flow showed patterns of introgressive hybridization in three morphologically ‘pure’ *V. japonicum* populations. We concluded that these differences in the genetic consequences of secondary contact are caused by historical colonization processes and/or natural selection.

## Availability of supporting data

The datasets supporting the results of this article are available in the Dryad (doi:10.5061/dryad.mg5rs).

## Additional file

Additional file 1: Figure S1. Map showing presence data for *V. atratum* (filled purple circles) and *V. japonicum* (filled yellow circles) populations used in niche modelling. **Figure S2.** Box-plots showing inter- and infra-population variation distribution in nine morphology character in each population. Rectangles define 25 % and 75 % quartiles (lower and upper edges of the box). Horizontal bar within box present the median. Whiskers extend up to the 90th percentiles and down to the 10th percentiles. Closed circle show outliers. **Figure S3.** Bar plot showing the inter- and infra-population variation pattern of hair density on leaf and corolla surface. **Figure S4.** Bayesian inference of the most likely number of clusters in the STRUCTURE analysis. (a) Plot of mean likelihood logarithmic probability of the data using 10 replicates runs at each value of *K* (*K*= 1-10). (b) Distribution of delta *K* for each *K* estimated from following Evanno et al. (2005). **Figure S5.** Deviance Index Criterion (DIC) for TESS analyses. **Table S1.** Details of the sample locations, sample size (*N*) and genetic diversity measure (*N*_a_: mean number of alleles per locus, *H*_E_: expected heterozygosity, *H*_O_: observed heterozygosity, AR: allele richness, PAR: private allele richness). **Table S2.** Description and references for the 10 nuclear SSR loci employed in this study. **Table S3.** Presence data used in niche modelling. **Table S4.** Bioclimatic variables obtained from the WorldClim and the variables used for predicting the distribution of *V. atratum* and *V. japonicum*. **Table S5.** Pollen fertility examined in the studied Vincetoxicum populations. Percentages of fertile pollen grains per population expressed as minimum, maximum and median. **Table S6.** Inbreeding coefficient for all populations at each microsatellite loci. **Table S7.** Linkage disequilibrium between all pairs of loci in each population. Significant *P*-values are given in bold after Bonferroni corrections (Rice 1989). **Table S8.** Pairwise estimates of genetic differentiation (*F*_ST_) between populations base on 10 microsatellite markers. **Table S9.** Assignment individuals based on result of STRUCTURE. **Table S10.** Estimates of gene flow (4*Nm*) among ‘pure’ *V. atratum* (A01-A07 and A09), ‘pure’ *V. japonicum* (J01-J08), introgressed *V. japonicum* (J09-J11), north group of putative hybrid populations (H01-H05) and south group of putative hybrid populations (H06 and H07). Source populations are listed by column, recipient populations listed by row. (DOCX 928 kb)
