# Supplementary material for: Long-term persisting hybrid swarm and geographic difference in hybridization pattern: genetic consequences of secondary contact between two Vincetoxicum species (Apocynaceae–Asclepiadoideae)
Source: BMC Evol Biol. 2016 Jan 22;16:20. doi: 10.1186/s12862-016-0587-2 (PMC4724111; doi:10.1186/s12862-016-0587-2)
Supplement: Additional file 1: Figure S1. — Map showing presence data for V. atratum (filled purple circles) and V. japonicum (filled yellow circles) populations used in niche modelling. Figure S2. Box-plots showing inter- and infra-population variation distribution in nine morphology character in each population. Rectangles define 25 % and 75 % quartiles (lower and upper edges of the box). Horizontal bar within box present the median. Whiskers extend up to the 90th percentiles and down to the 10th percentiles. Closed circle show outliers. Figure S3. Bar plot showing the inter- and infra-population variation pattern of hair density on leaf and corolla surface. Figure S4. Bayesian inference of the most likely number of clusters in the STRUCTURE analysis. (a) Plot of mean likelihood logarithmic probability of the data using 10 replicates runs at each value of K (K= 1-10). (b) Distribution of delta K for each K estimated from following Evanno et al. (2005). Figure S5. Deviance Index Criterion (DIC) for TESS analyses. Table S1. Details of the sample locations, sample size (N) and genetic diversity measure (N a: mean number of alleles per locus, H E: expected heterozygosity, H O: observed heterozygosity, AR: allele richness, PAR: private allele richness). Table S2. Description and references for the 10 nuclear SSR loci employed in this study. Table S3. Presence data used in niche modelling. Table S4. Bioclimatic variables obtained from the WorldClim and the variables used for predicting the distribution of V. atratum and V. japonicum. Table S5. Pollen fertility examined in the studied Vincetoxicum populations. Percentages of fertile pollen grains per population expressed as minimum, maximum and median. Table S6. Inbreeding coefficient for all populations at each microsatellite loci. Table S7. Linkage disequilibrium between all pairs of loci in each population. Significant P-values are given in bold after Bonferroni corrections (Rice 1989). Table S8. Pairwise estimates of genetic differentiation (F ST) between popu [file 12862_2016_587_MOESM1_ESM.docx]

**Supplementary Figure legends**

Figure S1 Map showing presence data for *V. atratum* (filled purple circles) and *V. japonicum* (filled yellow circles) populations used in niche modelling.

Figure S2 Box-plots showing inter- and infra-population variation distribution in nine morphology character in each population. Rectangles define 25% and 75% quartiles (lower and upper edges of the box). Horizontal bar within box present the median. Whiskers extend up to the 90th percentiles and down to the 10th percentiles. Closed circle show outliers.

Figure S3 Bar plot showing the inter- and infra-population variation pattern of hair density on leaf and corolla surface.

Figure S4 Bayesian inference of the most likely number of clusters in the STRUCTURE analysis. (a) Plot of mean likelihood logarithmic probability of the data using 10 replicates runs at each value of *K* (*K*= 1-10). (b) Distribution of delta *K* for each *K* estimated from following Evanno et al. (2005).

Figure S5 Deviance Index Criterion (DIC) for TESS analyses.

Fig. S1


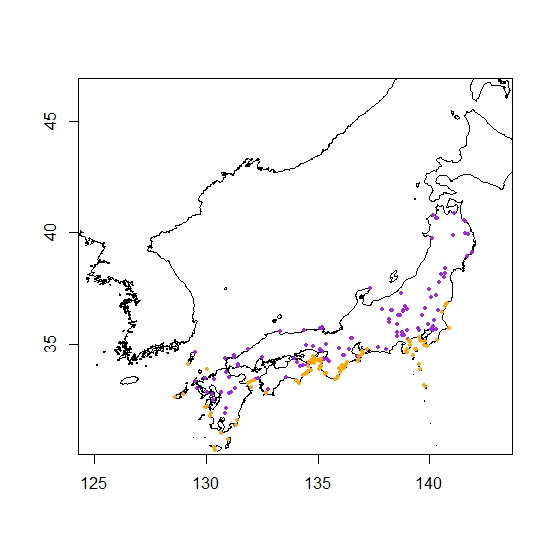


Fig. S2


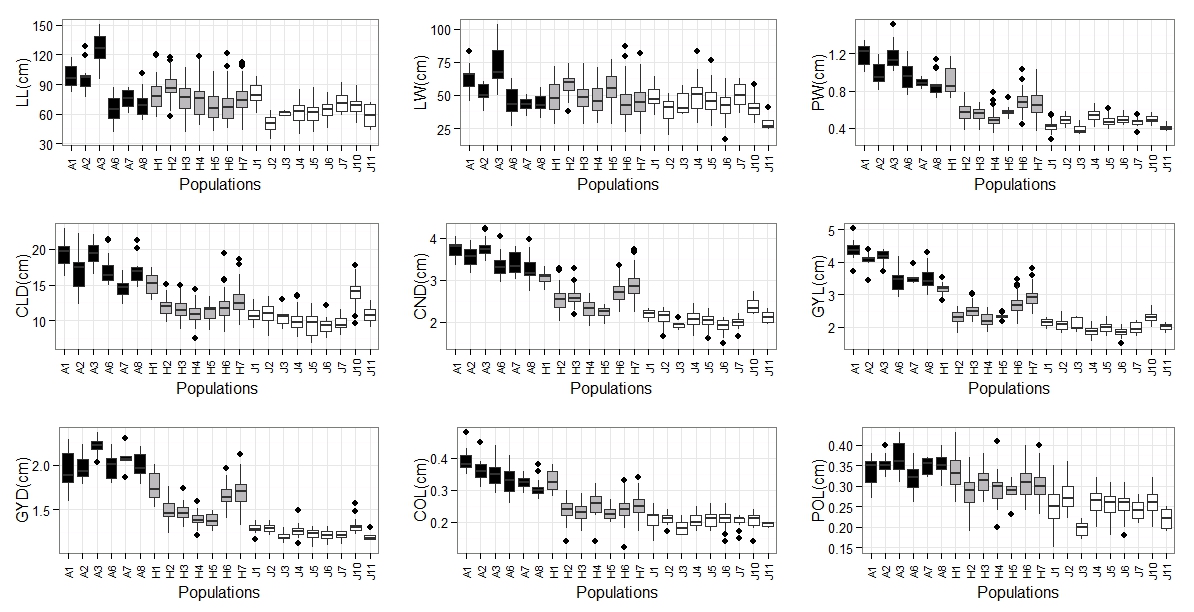


Fig. S3


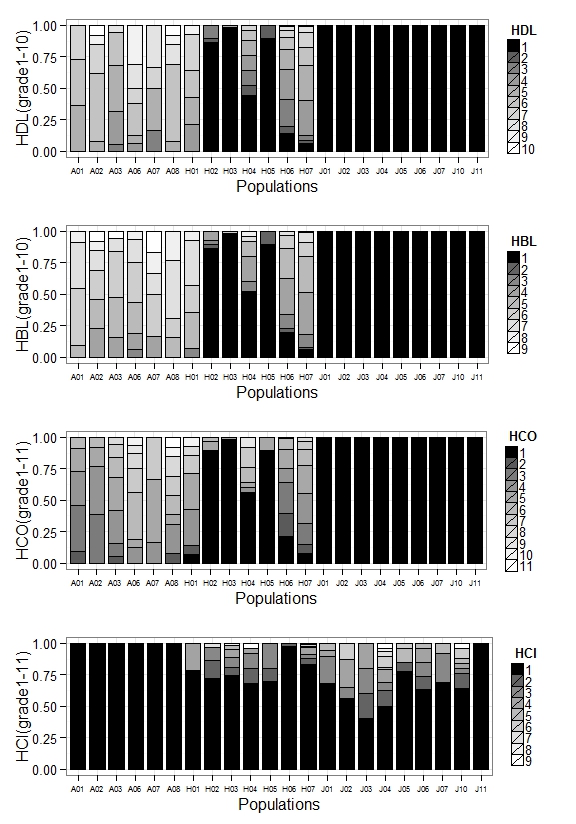


Fig. S4


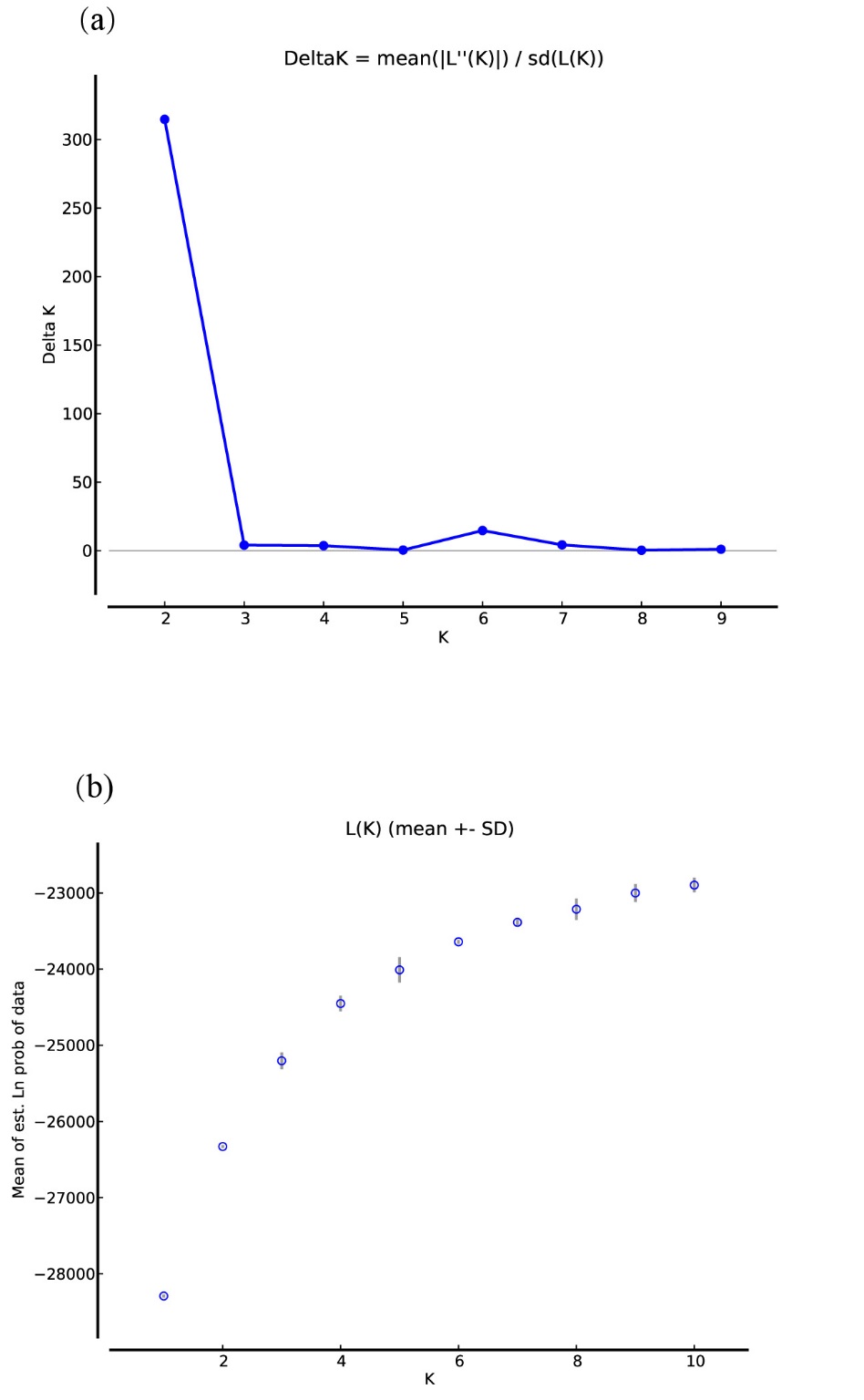


Fig. S5


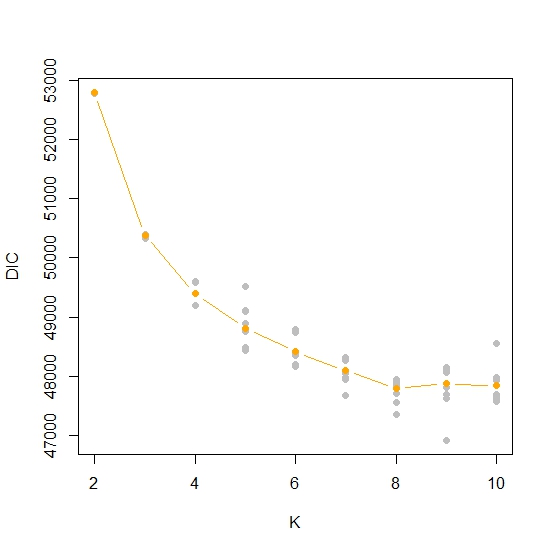


**Supplementary Table legends**

Table S1 Details of the sample locations, sample size (*N*) and genetic diversity measure (*N*_a_: mean number of alleles per locus, *H*_E_: expected heterozygosity, *H*_O_: observed heterozygosity, AR: allele richness, PAR: private allele richness).

Table S2 Description and references for the 10 nuclear SSR loci employed in this study.

Table S3 Presence data used in niche modelling.

Table S4 Bioclimatic variables obtained from the WorldClim and the variables used for predicting the distribution of *V. atratum* and *V. japonicum*.

Table S5 Pollen fertility examined in the studied Vincetoxicum populations. Percentages of fertile pollen grains per population expressed as minimum, maximum and median.

Table S6 Inbreeding coefficient for all populations at each microsatellite loci.

Table S7 Linkage disequilibrium between all pairs of loci in each population. Significant *P*-values are given in bold after Bonferroni corrections (Rice 1989).

Table S8 Pairwise estimates of genetic differentiation (*F*_ST_) between populations base on 10 microsatellite markers.

Table S9 Assignment individuals based on result of STRUCTURE

Table S10 Estimates of gene flow (4*Nm*) among 'pure' *V. atratum* (A01-A07 and A09), 'pure' *V. japonicum* (J01-J08), introgressed *V. japonicum* (J09-J11), north group of putative hybrid populations (H01-H05) and south group of putative hybrid populations (H06 and H07). Source populations are listed by column, recipient populations listed by row.

Table S1 Details of the sample locations, sample size (*N*) and genetic diversity measure (*N*_a_: mean number of alleles per locus, *H*_E_: expected heterozygosity, *H*_O_: observed heterozygosity, AR: allele richness, PAR: private allele richness).

| Population code | Localities | Latitude(N) | Longitude(E) | Altitude(m) | Type infered from  morphology and location | morphology (*N*) | SSR (*N*) | pollen fertility | *N*_a_ | *H*o | *H_E_* | AR | PAR |
| --- | --- | --- | --- | --- | --- | --- | --- | --- | --- | --- | --- | --- | --- |
| A01 | Tanesashi | 40.54 | 141.58 | 5 | *V. atratum* | 11 | 13 | 5 | 6.00 | 0.654(0.059) | 0.667(0.054) | 4.22 | 0.2 |
| A02 | Seaburi | 37.47 | 139.99 | 820 | *V. atratum* | 13 | 16 | 5 | 7.70 | 0.688(0.088) | 0.694(0.084) | 4.72 | 0.3 |
| A03 | Fuji | 35.40 | 138.79 | 1430 | *V. atratum* | 19 | 19 | 5 | 8.10 | 0.658(0.069) | 0.697(0.068) | 4.82 | 0.37 |
| A04 | Soni | 34.52 | 136.16 | 680 | *V. atratum* | 0 | 6 | 0 | 4.70 | 0.650(0.107) | 0.618(0.092) | 4.37 | 0.17 |
| A05 | Tango | 35.78 | 135.22 | 90 | *V. atratum* | 0 | 16 | 0 | 5.60 | 0.619(0.093) | 0.611(0.082) | 3.89 | 0.14 |
| A06 | Tsunoshima | 34.36 | 130.84 | 6 | *V. atratum* | 16 | 15 | 0 | 6.90 | 0.726(0.061) | 0.663(0.055) | 4.45 | 0.25 |
| A07 | Hiraodai | 33.76 | 130.90 | 390 | *V. atratum* | 4 | 4 | 0 | 4.30 | 0.692(0.097) | 0.597(0.082) | 4.30 | 0.53 |
| A08 | Kawauchitouge | 33.34 | 129.53 | 220 | *V. atratum* | 13 | 0 | 0 | — | — | — | — | — |
| A09 | Aso | 32.81 | 131.03 | 550 | *V. atratum* | 0 | 10 | 0 | 4.30 | 0.720(0.096) | 0.557(0.073) | 3.37 | 0.01 |
| H01 | Karakuwa | 38.86 | 141.67 | 10 | *intermediate* | 13 | 17 | 5 | 6.20 | 0.659(0.079) | 0.652(0.064) | 4.22 | 0.13 |
| H02 | Oshika | 38.30 | 141.54 | 10 | *intermediate* | 28 | 28 | 5 | 6.30 | 0.617(0.053) | 0.612(0.058) | 3.70 | 0.12 |
| H03 | Enoshima | 38.40 | 141.60 | 10 | *intermediate* | 62 | 62 | 5 | 7.40 | 0.635(0.062) | 0.622(0.061) | 3.70 | 0.09 |
| H04 | Kinkasan | 38.29 | 141.58 | 10 | *intermediate* | 24 | 26 | 5 | 5.90 | 0.623(0.07) | 0.607(0.067) | 3.77 | 0.04 |
| H05 | Ajishima | 38.28 | 141.48 | 30 | *intermediate* | 10 | 14 | 5 | 4.20 | 0.603(0.087) | 0.519(0.072) | 3.11 | 0.01 |
| H06 | Matsushima | 36.26 | 136.15 | 5 | *intermediate* | 97 | 97 | 5 | 10.20 | 0.719(0.059) | 0.747(0.058) | 4.87 | 0.26 |
| H07 | Oshima | 36.25 | 136.12 | 4 | *intermediate* | 94 | 94 | 5 | 10.80 | 0.691(0.067) | 0.691(0.066) | 4.55 | 0.20 |
| J01 | Itsuura | 36.83 | 140.80 | 10 | *V. japonicum* | 19 | 19 | 5 | 6.40 | 0.589(0.074) | 0.654(0.062) | 4.12 | 0.28 |
| J02 | Inubou | 35.71 | 140.87 | 3 | *V. japonicum* | 23 | 23 | 5 | 7.20 | 0.674(0.073) | 0.654(0.062) | 4.26 | 0.09 |
| J03 | Katsura | 35.16 | 140.33 | 3 | *V. japonicum* | 3 | 3 | 5 | 3.10 | 0.633(0.136) | 0.483(0.099) | 3.10 | 0.25 |
| J04 | Nojimazaki | 34.90 | 139.89 | 10 | *V. japonicum* | 48 | 53 | 0 | 8.90 | 0.662(0.069) | 0.672(0.061) | 4.21 | 0.22 |
| J05 | Shirahama | 34.69 | 138.97 | nearly 0 | *V. japonicum* | 27 | 27 | 0 | 7.90 | 0.641(0.055) | 0.666(0.051) | 4.12 | 0.26 |
| J06 | Ooshima | 34.79 | 139.39 | 20 | *V. japonicum* | 26 | 27 | 0 | 8.50 | 0.626(0.068) | 0.667(0.065) | 4.34 | 0.52 |
| J07 | Toshima | 34.53 | 139.28 | 10 | *V. japonicum* | 13 | 15 | 0 | 5.70 | 0.560(0.084) | 0.633(0.068) | 3.94 | 0.17 |
| J08 | Hachijyoushima | 33.12 | 139.82 | 10 | *V. japonicum* | 0 | 11 | 0 | 2.60 | 0.345(0.102) | 0.289(0.075) | 2.06 | 0.02 |
| J09 | Irakosaki | 34.58 | 137.02 | 15 | *V. japonicum* | 0 | 22 | 0 | 7.20 | 0.645(0.078) | 0.673(0.072) | 4.33 | 0.17 |
| J10 | Yuki | 33.77 | 134.59 | 20 | *V. japonicum* | 25 | 26 | 0 | 7.90 | 0.562(0.087) | 0.644(0.07) | 4.23 | 0.52 |
| J11 | Mugi | 33.67 | 134.42 | 8 | *V. japonicum* | 4 | 8 | 0 | 5.20 | 0.638(0.06) | 0.642(0.061) | 4.22 | 0.17 |

Table S2 Description and references for the 10 nuclear SSR loci employed in this study.

| Locus | Lable ^a^ | multiplex set | reference |
| --- | --- | --- | --- |
| Vkat2 ^b^ | pet | 1 | Yamashiro et al., unpublished |
| Vkat3 ^c^ | ned | 1 | Yamashiro et al., unpublished |
| vinc5 | fam | 3 | Tada et al. 2009 |
| vinc107 | vic | 3 | Tada et al. 2009 |
| vinc118 | ned | 3 | Tada et al. 2009 |
| Vpy 012 | ned | 2 | Nakahama et al. 2012 |
| Vpy 013 | vic | 1 | Nakahama et al. 2012 |
| Vpy 016 | fam | 2 | Nakahama et al. 2012 |
| Vpy 018 | vic | 2 | Nakahama et al. 2012 |
| Vpy 022 | fam | 1 | Nakahama et al. 2012 |

^a^ Fluorescent dye used to label the 5’-end of each forward primer, respetively.

^b^ primer sequence of Vkat2: Forward: 5’-TGTAATATTGCCCATAAGGGGG-3’, Reverse: 5’-TTAAGCTTGACGAGCTTGTGC.

^c^ primer sequence of Vkat3: Forward: 5’-AAGGGCATGAAGAATTAAGACCCG-3, Reverse: 5’AAAAGTTCAGTGAGTGAACCGAGG-3’.

Table S3 Presence data used in niche modelling.

| species | Longitude (E) | Latitude (N) |
| --- | --- | --- |
| *V. atratum* | 131.24439 | 34.431398 |
| *V. atratum* | 131.87061 | 34.758247 |
| *V. atratum* | 133.32514 | 35.568251 |
| *V. atratum* | 140.5229 | 38.126176 |
| *V. atratum* | 140.70027 | 38.198116 |
| *V. atratum* | 131.28067 | 34.5113 |
| *V. atratum* | 138.80664 | 35.552554 |
| *V. atratum* | 140.31177 | 35.659463 |
| *V. atratum* | 132.50315 | 34.416969 |
| *V. atratum* | 141.91996 | 39.104926 |
| *V. atratum* | 138.74706 | 37.287955 |
| *V. atratum* | 140.71052 | 38.411336 |
| *V. atratum* | 140.65482 | 37.987644 |
| *V. atratum* | 138.19285 | 35.991554 |
| *V. atratum* | 138.18426 | 36.101202 |
| *V. atratum* | 138.59659 | 36.282203 |
| *V. atratum* | 137.90375 | 36.565017 |
| *V. atratum* | 136.51101 | 35.283979 |
| *V. atratum* | 140.21456 | 36.091029 |
| *V. atratum* | 138.99508 | 36.591231 |
| *V. atratum* | 130.68163 | 32.832334 |
| *V. atratum* | 139.50992 | 35.717371 |
| *V. atratum* | 133.59157 | 33.541672 |
| *V. atratum* | 132.77671 | 32.981002 |
| *V. atratum* | 139.56153 | 35.622291 |
| *V. atratum* | 134.08973 | 34.174945 |
| *V. atratum* | 134.20245 | 34.028485 |
| *V. atratum* | 134.78172 | 34.907972 |
| *V. atratum* | 136.39825 | 34.776383 |
| *V. atratum* | 140.30286 | 37.20443 |
| *V. atratum* | 141.69997 | 38.956162 |
| *V. atratum* | 141.13015 | 40.867045 |
| *V. atratum* | 140.38711 | 36.542276 |
| *V. atratum* | 129.49414 | 34.665298 |
| *V. atratum* | 132.19766 | 33.432962 |
| *V. atratum* | 135.52821 | 34.24863 |
| *V. atratum* | 133.93854 | 34.360547 |
| *V. atratum* | 140.33031 | 40.631931 |
| *V. atratum* | 140.14064 | 39.738526 |
| *V. atratum* | 141.76171 | 39.933004 |
| *V. atratum* | 140.06998 | 35.688967 |
| *V. atratum* | 136.52449 | 35.264033 |
| *V. atratum* | 131.44003 | 34.033864 |
| *V. atratum* | 131.01841 | 33.538871 |
| *V. atratum* | 130.33067 | 32.488957 |
| *V. atratum* | 130.2775 | 32.769854 |
| *V. atratum* | 130.90239 | 33.759908 |
| *V. atratum* | 141.60831 | 39.972276 |
| *V. atratum* | 137.33047 | 37.511316 |
| *V. atratum* | 139.03657 | 35.217653 |
| *V. atratum* | 138.8583 | 35.414505 |
| *V. atratum* | 130.36844 | 33.436086 |
| *V. atratum* | 129.61304 | 33.046468 |
| *V. atratum* | 130.06692 | 32.857408 |
| *V. atratum* | 130.91286 | 32.125224 |
| *V. atratum* | 131.13098 | 32.843472 |
| *V. atratum* | 130.86297 | 31.916584 |
| *V. atratum* | 132.32872 | 33.499217 |
| *V. atratum* | 135.97221 | 34.801783 |
| *V. atratum* | 135.09114 | 35.734717 |
| *V. atratum* | 135.99361 | 33.861627 |
| *V. atratum* | 136.78458 | 34.462317 |
| *V. atratum* | 134.37484 | 35.611827 |
| *V. atratum* | 135.18048 | 34.702199 |
| *V. atratum* | 135.10775 | 34.773288 |
| *V. atratum* | 136.16661 | 34.519168 |
| *V. atratum* | 139.01409 | 35.638626 |
| *V. atratum* | 141.60338 | 40.515845 |
| *V. atratum* | 135.39778 | 34.980173 |
| *V. atratum* | 135.24684 | 34.331437 |
| *V. atratum* | 135.29339 | 34.314442 |
| *V. atratum* | 135.1187 | 34.284086 |
| *V. atratum* | 135.43445 | 34.348313 |
| *V. atratum* | 134.47215 | 34.940007 |
| *V. atratum* | 135.11916 | 34.733576 |
| *V. atratum* | 134.51991 | 34.060504 |
| *V. atratum* | 134.33205 | 34.05302 |
| *V. atratum* | 141.58 | 40.54 |
| *V. atratum* | 139.99 | 37.47 |
| *V. atratum* | 138.79 | 35.4 |
| *V. atratum* | 136.16 | 34.52 |
| *V. atratum* | 135.22 | 35.78 |
| *V. atratum* | 130.84 | 34.36 |
| *V. atratum* | 130.9 | 33.76 |
| *V. atratum* | 129.53 | 33.34 |
| *V. atratum* | 131.03 | 32.81 |
| *V. atratum* | 140.1875 | 35.791667 |
| *V. atratum* | 140.1875 | 35.791667 |
| *V. atratum* | 140.1875 | 35.625 |
| *V. atratum* | 140.1875 | 35.625 |
| *V. atratum* | 140.1875 | 35.625 |
| *V. atratum* | 139.9375 | 35.875 |
| *V. atratum* | 139.9375 | 35.875 |
| *V. atratum* | 138.32648 | 36.537 |
| *V. atratum* | 138.3125 | 36.541667 |
| *V. atratum* | 139.6875 | 36.291667 |
| *V. atratum* | 138.5625 | 35.375 |
| *V. atratum* | 138.5625 | 35.375 |
| *V. atratum* | 137.6875 | 34.875 |
| *V. atratum* | 137.6875 | 34.875 |
| *V. atratum* | 138.5625 | 35.375 |
| *V. atratum* | 140.0625 | 37.125 |
| *V. atratum* | 140.0625 | 37.125 |
| *V. atratum* | 140.0625 | 37.125 |
| *V. atratum* | 140.1875 | 40.791667 |
| *V. atratum* | 131.3125 | 33.041667 |
| *V. atratum* | 138.5625 | 35.541667 |
| *V. atratum* | 138.8125 | 36.541667 |
| *V. atratum* | 129.9375 | 33.458333 |
| *V. atratum* | 139.9375 | 35.875 |
| *V. atratum* | 138.0625 | 34.791667 |
| *V. atratum* | 135.1875 | 35.708333 |
| *V. atratum* | 138.8125 | 36.458333 |
| *V. atratum* | 131.4375 | 34.125 |
| *V. atratum* | 138.5625 | 35.875 |
| *V. atratum* | 140.3125 | 40.625 |
| *V. atratum* | 131.3125 | 31.375 |
| *V. atratum* | 140.4375 | 37.791667 |
| *V. atratum* | 138.9375 | 36.708333 |
| *V. atratum* | 138.3125 | 36.541667 |
| *V. atratum* | 139.6875 | 36.291667 |
| *V. atratum* | 141.0625 | 39.875 |
| *V. atratum* | 138.6875 | 36.291667 |
| *V. atratum* | 138.6875 | 36.291667 |
| *V. atratum* | 139.8125 | 36.625 |
| *V. japonicum* | 140.757085 | 36.796527 |
| *V. japonicum* | 140.727903 | 36.725065 |
| *V. japonicum* | 140.869406 | 35.708171 |
| *V. japonicum* | 139.831047 | 35.145511 |
| *V. japonicum* | 139.755645 | 34.972379 |
| *V. japonicum* | 139.608746 | 35.192187 |
| *V. japonicum* | 139.652648 | 35.140563 |
| *V. japonicum* | 139.554304 | 35.297969 |
| *V. japonicum* | 138.977682 | 34.667962 |
| *V. japonicum* | 138.98723 | 34.659102 |
| *V. japonicum* | 138.964485 | 34.653348 |
| *V. japonicum* | 139.349602 | 34.761164 |
| *V. japonicum* | 139.749611 | 33.150528 |
| *V. japonicum* | 139.557007 | 34.096494 |
| *V. japonicum* | 137.035827 | 34.578235 |
| *V. japonicum* | 137.230549 | 34.788492 |
| *V. japonicum* | 137.001028 | 34.673387 |
| *V. japonicum* | 136.981866 | 34.543601 |
| *V. japonicum* | 136.865673 | 34.464557 |
| *V. japonicum* | 136.33129 | 34.189049 |
| *V. japonicum* | 136.186623 | 33.940963 |
| *V. japonicum* | 136.80455 | 34.269164 |
| *V. japonicum* | 136.813 | 34.230863 |
| *V. japonicum* | 136.115384 | 33.893242 |
| *V. japonicum* | 136.098904 | 33.884193 |
| *V. japonicum* | 136.02377 | 33.765198 |
| *V. japonicum* | 136.294995 | 34.096502 |
| *V. japonicum* | 135.933175 | 33.578371 |
| *V. japonicum* | 135.862355 | 33.471784 |
| *V. japonicum* | 135.332265 | 33.67145 |
| *V. japonicum* | 135.078699 | 33.890766 |
| *V. japonicum* | 135.335267 | 33.692825 |
| *V. japonicum* | 135.148958 | 34.011338 |
| *V. japonicum* | 135.100328 | 34.003532 |
| *V. japonicum* | 135.141526 | 34.189733 |
| *V. japonicum* | 135.393955 | 33.710944 |
| *V. japonicum* | 135.095296 | 34.314552 |
| *V. japonicum* | 135.149713 | 34.326673 |
| *V. japonicum* | 135.152985 | 34.330629 |
| *V. japonicum* | 135.024894 | 34.293015 |
| *V. japonicum* | 134.932733 | 34.299468 |
| *V. japonicum* | 134.870386 | 34.308924 |
| *V. japonicum* | 134.798082 | 34.449074 |
| *V. japonicum* | 134.820776 | 34.17255 |
| *V. japonicum* | 134.714702 | 34.216907 |
| *V. japonicum* | 134.619042 | 34.185278 |
| *V. japonicum* | 134.588397 | 34.03211 |
| *V. japonicum* | 134.698561 | 33.833676 |
| *V. japonicum* | 134.550332 | 33.737015 |
| *V. japonicum* | 134.517458 | 33.706318 |
| *V. japonicum* | 134.428323 | 33.663787 |
| *V. japonicum* | 134.363135 | 33.603902 |
| *V. japonicum* | 134.173084 | 33.246786 |
| *V. japonicum* | 134.111308 | 33.294454 |
| *V. japonicum* | 134.039296 | 33.374339 |
| *V. japonicum* | 132.015022 | 33.343835 |
| *V. japonicum* | 132.152437 | 33.374088 |
| *V. japonicum* | 130.034193 | 33.868802 |
| *V. japonicum* | 129.203921 | 34.105959 |
| *V. japonicum* | 129.969033 | 32.182664 |
| *V. japonicum* | 131.900269 | 33.266653 |
| *V. japonicum* | 132.000205 | 33.057015 |
| *V. japonicum* | 128.620461 | 32.623803 |
| *V. japonicum* | 128.992005 | 32.770694 |
| *V. japonicum* | 130.020653 | 32.179689 |
| *V. japonicum* | 130.02988 | 32.202279 |
| *V. japonicum* | 131.340908 | 31.362364 |
| *V. japonicum* | 131.411546 | 31.579784 |
| *V. japonicum* | 129.970979 | 32.17954 |
| *V. japonicum* | 130.190519 | 31.755107 |
| *V. japonicum* | 130.414054 | 30.266699 |
| *V. japonicum* | 130.382383 | 30.392197 |
| *V. japonicum* | 130.993362 | 30.72933 |
| *V. japonicum* | 130.22007 | 31.928945 |
| *V. japonicum* | 130.683402 | 31.024295 |
| *V. japonicum* | 129.539004 | 29.913132 |
| *V. japonicum* | 129.614413 | 28.476496 |
| *V. japonicum* | 129.589264 | 28.46748 |
| *V. japonicum* | 129.320357 | 28.347404 |
| *V. japonicum* | 128.881933 | 27.724065 |
| *V. japonicum* | 140.8 | 36.83 |
| *V. japonicum* | 140.87 | 35.71 |
| *V. japonicum* | 140.33 | 35.16 |
| *V. japonicum* | 139.89 | 34.9 |
| *V. japonicum* | 138.97 | 34.69 |
| *V. japonicum* | 139.39 | 34.79 |
| *V. japonicum* | 139.28 | 34.53 |
| *V. japonicum* | 139.82 | 33.12 |
| *V. japonicum* | 137.02 | 34.58 |
| *V. japonicum* | 134.59 | 33.77 |
| *V. japonicum* | 134.42 | 33.67 |
| *V. japonicum* | 139.115591 | 35.157458 |
| *V. japonicum* | 139.140588 | 35.149128 |
| *V. japonicum* | 139.61554 | 35.132466 |
| *V. japonicum* | 139.61554 | 35.132466 |
| *V. japonicum* | 139.653037 | 35.140798 |
| *V. japonicum* | 139.604878 | 33.878756 |
| *V. japonicum* | 139.592655 | 33.895143 |
| *V. japonicum* | 139.140588 | 35.149128 |
| *V. japonicum* | 139.153087 | 35.149129 |
| *V. japonicum* | 139.088667 | 35.167194 |
| *V. japonicum* | 135.982998 | 33.936405 |
| *V. japonicum* | 138.965274 | 34.659614 |
| *V. japonicum* | 139.009528 | 34.798658 |
| *V. japonicum* | 139.126806 | 34.969194 |
| *V. japonicum* | 140.58992 | 36.45253 |
| *V. japonicum* | 136.11186 | 34.05186 |
| *V. japonicum* | 136.11186 | 34.05186 |
| *V. japonicum* | 136.2469 | 34.0174 |
| *V. japonicum* | 128.3125 | 26.875 |
| *V. japonicum* | 138.98981 | 34.65428 |
| *V. japonicum* | 138.965 | 34.6518 |
| *V. japonicum* | 138.9757 | 34.6687 |
| *V. japonicum* | 135.76981 | 33.44519 |
| *V. japonicum* | 128.3125 | 26.875 |
| *V. japonicum* | 128.5625 | 27.375 |
| *V. japonicum* | 128.4375 | 27.041667 |
| *V. japonicum* | 128.4375 | 27.041667 |
| *V. japonicum* | 128.4375 | 27.041667 |
| *V. japonicum* | 136.8752 | 34.3302 |
| *V. japonicum* | 135.921111 | 33.580278 |
| *V. japonicum* | 139.61554 | 35.157464 |
| *V. japonicum* | 135.34828 | 33.68136 |
| *V. japonicum* | 134.7 | 34.283333 |
| *V. japonicum* | 134.816667 | 34.283333 |
| *V. japonicum* | 132.6833333 | 32.86666667 |
| *V. japonicum* | 132.7 | 32.81666667 |
| *V. japonicum* | 134.0333333 | 33.36666667 |

Table S4 Bioclimatic variables obtained from the WorldClim and the variables used for predicting the distribution of *V. atratum* and *V. japonicum*.

| Code | Bioclimatic variable | *V. atratum* | *V. japonicum* |
| --- | --- | --- | --- |
| BIO1 | Annual Mean Temperature |  |  |
| BIO2 | Mean Diurnal Range [Mean of monthly (max temp–min temp)] |  |  |
| BIO3 | Isothermality (BIO2/BIO7) (* 100) | √ |  |
| BIO4 | Temperature Seasonality (standard deviation * 100) | √ |  |
| BIO5 | Max Temperature of Warmest Month |  | √ |
| BIO6 | Min Temperature of Coldest Month |  |  |
| BIO7 | Temperature Annual Range (BIO5-BIO6) |  |  |
| BIO8 | Mean Temperature of Wettest Quarter | √ |  |
| BIO9 | Mean Temperature of Driest Quarter |  |  |
| BIO10 | Mean Temperature of Warmest Quarter | √ |  |
| BIO11 | Mean Temperature of Coldest Quarter |  | √ |
| BIO12 | Annual Precipitation |  |  |
| BIO13 | Precipitation of Wettest Month |  |  |
| BIO14 | Precipitation of Driest Month |  |  |
| BIO15 | Precipitation Seasonality (Coefficient of Variation) | √ |  |
| BIO16 | Precipitation of Wettest Quarter |  |  |
| BIO17 | Precipitation of Driest Quarter |  | √ |
| BIO18 | Precipitation of Warmest Quarter | √ | √ |
| BIO19 | Precipitation of Coldest Quarter | √ | √ |

Table S5 Pollen fertility examined in the studied *Vincetoxicum* populations. Percentages of fertile pollen grains per population expressed as minimum, maximum and median.

| Population | Min-max | Median |
| --- | --- | --- |
| A01 | 97.00-100.00 | 99.00 |
| A02 | 100.00-100.00 | 100.00 |
| A03 | 100.00-100.00 | 100.00 |
| H01 | 100.00-100.00 | 100.00 |
| H02 | 100.00-100.00 | 100.00 |
| H03 | 98.00-100.00 | 99.60 |
| H04 | 100.00-100.00 | 100.00 |
| H05 | 98.00-100.00 | 99.60 |
| H06 | 96.00-100.00 | 99.20 |
| H07 | 96.00-100.00 | 99.20 |
| J01 | 100.00-100.00 | 100.00 |
| J02 | 100.00-100.00 | 100.00 |
| J03 | 100.00-100.00 | 100.00 |

Table S6 Inbreeding coefficient for all populations at each microsatellite loci.

|  |  |  |  |  | SSR loci |  |  |  |  |  |
| --- | --- | --- | --- | --- | --- | --- | --- | --- | --- | --- |
| population | Vpy 016 | Vpy 018 | vinc3-1 | Vpy 022 | Vpy 013 | kaito2 | Vpy 012 | vinc5 | vinc107 | vinc118 |
| A01 | 0.176 | 0.032 | 0.200 | -0.519 | 0.139 | 0.019 | 0.204 | -0.114 | 0.185 | 0.193* |
| A02 | 0.056 | 0.147 | — | 0.202 | -0.099 | 0.130 | 0.089 | -0.062 | -0.017 | -0.025 |
| A03 | -0.044 | 0.219 | -0.059 | 0.182* | -0.029 | -0.019 | 0.073 | 0.145 | 0.086 | 0.215** |
| A04 | 0.057 | -0.042 | — | 0 | -0.277 | 0.091 | 0.216 | 0.400* | -0.304 | 0.130 |
| A05 | -0.080 | -0.132 | — | -0.066 | 0.083 | 0.032 | 0.298* | 0.297 | -0.046 | -0.169 |
| A06 | 0.024 | -0.020 | -0.128 | -0.260 | -0.071 | -0.110 | 0.105 | -0.012 | -0.152 | -0.046 |
| A07 | 0.217 | -0.286 | — | -0.091 | -0.200 | -0.143 | 0.250 | -0.286 | 0.273 | 0 |
| A09 | -0.213 | -0.514 | — | -0.370 | -0.295 | -0.165 | -0.139 | -0.191 | 0 | -0.333 |
| H01 | 0.349* | -0.070 | -0.063 | 0.319 | 0.097 | -0.049 | 0.020 | -0.166 | -0.012 | 0.045 |
| H02 | -0.101 | 0.075 | 0.029 | -0.28 | -0.194 | -0.008 | 0.332 | -0.018 | 0.031 | -0.045 |
| H03 | 0.239 | -0.049 | 0.055 | -0.144 | 0.041 | 0.042 | 0.081 | -0.136 | -0.016 | -0.185 |
| H04 | 0.320 | 0.079 | -0.117 | -0.105 | -0.112 | 0.075 | 0.207 | -0.113 | -0.065 | -0.070 |
| H05 | -0.020 | -0.164 | -0.234 | -0.095 | -0.071 | -0.040 | -0.035 | -0.102 | -0.300 | -0.072 |
| H06 | 0.269*** | 0.054** | -0.078 | -0.039 | -0.048 | 0.001 | 0.201 | -0.026 | -0.058 | 0.070*** |
| H07 | -0.059 | -0.031 | -0.046 | 0.118 | 0.029 | -0.017 | 0.024 | 0.002 | 0.033 | 0.044 |
| J01 | 0.339* | 0.534*** | 0.043 | -0.059 | 0.077 | 0.178* | 0.257 | -0.059 | 0.144* | -0.312 |
| J02 | -0.017 | -0.130 | -0.103 | 0.279* | -0.046 | -0.073 | 0.047 | 0.218** | -0.004 | -0.075 |
| J03 | 0 | 0.111 | -0.200 | — | -0.091 | -0.091 | -0.333 | 0 | -0.200 | — |
| J04 | 0.145** | -0.071 | -0.104 | 0.043 | 0.144 | -0.023 | 0.018 | -0.035 | -0.006 | 0.216* |
| J05 | 0.236 | 0.065 | 0.134* | -0.156 | 0.034 | 0.063 | -0.038 | -0.067 | 0.229* | -0.015 |
| J06 | 0.163 | -0.025 | 0.007 | -0.083 | -0.039 | -0.011 | 0.055* | 0.480*** | -0.004 | 0.102 |
| J07 | -0.082 | -0.046 | 0.058* | 1 | -0.093 | 0.308** | 0.046 | 0.545*** | 0.197 | 0.229 |
| J08 | — | 0.341* | -0.250 | — | -0.538 | -0.136 | -0.429 | — | 0.180 | 0 |
| J09 | 0.116 | 0.149 | -0.146 | — | -0.139 | 0.041 | -0.071 | 0.047 | 0.336* | 0.267 |
| J10 | 0.216* | 0.007 | 0.058 | 0.275 | 0.094 | 0.012 | 0.087 | 0.242* | 0.468* | 0.457* |
| J11 | 0.097 | -0.021 | 0.195 | -0.273 | 0.195 | -0.065 | 0.243 | 0.255* | -0.037 | -0.091 |

Table S9 Assignment individuals based on result of STRUCTURE.

|  | *V. atratum* | *V. japonicum* | admixture |
| --- | --- | --- | --- |
| A01 | 12 | 0 | 1 |
| A02 | 14 | 0 | 2 |
| A03 | 17 | 0 | 2 |
| A04 | 6 | 0 | 0 |
| A05 | 16 | 0 | 0 |
| A06 | 15 | 0 | 0 |
| A07 | 4 | 0 | 0 |
| A09 | 10 | 0 | 0 |
| H01 | 1 | 3 | 13 |
| H02 | 0 | 27 | 2 |
| H03 | 0 | 61 | 1 |
| H04 | 0 | 22 | 4 |
| H05 | 0 | 14 | 0 |
| H06 | 92 | 0 | 5 |
| H07 | 76 | 1 | 17 |
| J01 | 0 | 17 | 2 |
| J02 | 0 | 19 | 4 |
| J03 | 0 | 3 | 0 |
| J04 | 0 | 51 | 1 |
| J05 | 0 | 21 | 6 |
| J06 | 0 | 26 | 1 |
| J07 | 0 | 14 | 1 |
| J08 | 0 | 10 | 1 |
| J09 | 7 | 1 | 14 |
| J10 | 18 | 0 | 8 |
| J11 | 3 | 1 | 4 |

Table S10 Estimates of gene flow (4*Nm*) among five groups. Source populations are listed by column, recipient populations listed by row.

|  | pure' *V. atratum* | north group of putative hybrid population | south group of putative hybrid population | pure' *V. japonicum* | introgressed *V. japonicum* |
| --- | --- | --- | --- | --- | --- |
| pure' *V. atratum* | — | *1.78* (1.03-2.41) | *4.37* (1.87-6.10) | *1.19* (0.26-1.70) | *1.26* (0.77-1.52) |
| north group of putative hybrid population | *4.06* (1.61-5.22) | — | *1.20* (0.79-1.79) | *1.69* (1.27-2.03) | *0.47* (0.14-1.05) |
| south group of putative hybrid population | *0.86* (0.38-1.27) | *4.71* (0.41-6.49) | — | *0.95* (0.86-7.68) | *0.85* (0.24-0.92) |
| pure' *V. japonicum* | *19.27* (10.97-30.80) | *4.35* (2.35-8.76) | *4.04* (3.26-8.68) | — | *12.22* (9.63-16.82) |
| introgressed *V. japonicum* | *0.63* (0.17-1.17) | *1.19* (0.37-2.31) | *0.97* (0.34-1.80) | *2.43* (1.82-2.81) | — |

MPE (most probable estimates) in italics and 95% credible intervals are in brackets.
